# Some Minor Rectal Lesions1Read at the thirty-eighth annual meeting of the Medical Association of Central New York, at Buffalo, October 24, 1905.

**Published:** 1906-04

**Authors:** Dwight H. Murray

**Affiliations:** Syracuse, N. Y.


### BUFFALO MEDICAL JOURNAL.

VOL. LXI. APRIL, 1906. No. 9

### ORIGINAL COMMUNICATIONS.

### Some Minor Rectal Lesions.[1]

BY DWIGHT H. MURRAY, M. D., Syracuse, N. Y.

IN the examination and treatment of ano-rectal diseases, pathological conditions exist that are overlooked by many good diagnosticians. This is my excuse, if one is needed, for a paper containing suggestions regarding some of the minor lesions.

Divulsion of the sphincter muscle, judging from statements of many physicians and patients with whom I come in contact, is not well understood. At least ten minutes, on an average, should be occupied in this procedure. The muscle should be carefully and slowly tired out, or paralysed, not quickly stretched and the muscular fibers torn, as may happen when rapidly done. I have had patients who gave a history of rapid dilatation of the sphincter, which resulted in a partial loss of sphincteric control. These patients never cease to condemn the operator; indeed, they are sometimes justified in so doing. Rectal operations done after a proper divulsion cause much less pain, and a soreness only is complained of in most cases.

Fissure ani is a most painful condition. A sharp cutting, tearing, or burning pain coming on during or after a bowel movement is indicative of this trouble. Patients do not always know just the time it began; they may first notice a little bleeding after stool, spasm of sphincter, and an increased constipation caused by fear of pain. The stool may then become dry and hard, making the fissure worse. These patients are really in a very nervous and distressed condition, with many direct and reflex. symptoms. Sometimes the only symptom of a fissure in ano is a pain in the heel, which is often treated for rheumatism, but it is well to remember that fissure ani is a possible cause.

When a fissure is recent, it may be treated successfully, without operation, if there is no spasm of the sphincter, and no induration. Orthoform applied to its base, followed by pure ichthyol,

1 Read at the thirty-eighth annual meeting of the Medical Association of Central New York, at Buffalo, October 24, 1905.

applied every other day, is a treatment best adapted for most non-operative cases. The average length of time to cure uncomplicated cases in this way, is from two to four weeks. With hypertrophied, hard, spasmodically contracted sphincter, and a sentinel pile well developed, we will not succeed without operative interference. Operative cases are treated by dilatation, incision and excision, which different methods are explained by their names.

The crypts of Morgagni are situated at the junction of the two sphincters, between the columns of Morgagni. If these become inflamed, or an accident occurs during the passage of stool which breaks the mucous membrane at the base of the crypt, it is easy for the edge of the mucous membrane to become undermined. A dissection occurs that often extends to the depth of half an inch or more. Fecal matter gravitates into these pockets and produces an irritation that is manifested by a variety of symptoms,—smarting, burning, many nervous reflexes, and sometimes severe pain. Diseased crypts are a frequent source of abscess when infected, and result, frequently, in fistulæ with which all are familiar. In nearly all these conditions we find a very tight sphincter.

These diseased crypts are nearly always tender, and are the cause, in many patients, of continued constipation. One has a call to attend toilet obligations, and finds that the fecal mass descends to, but he is unable to pass it by, the sphincter, which closes spasmodically upon the approach of a foreign body. After the passage of stool an irritation remains which makes the patient very uncomfortable, sometimes in severe pain for hours. If fecal matter has been forced into the depth of the pocket, there will be alternate spasm and relaxation of the sphincter, until the crypt is cleared of all foreign material.

Situated at the entrance of these diseased crypts, when patients have been long afflicted, we occasionally find hypertrophied papules, sometimes of considerable size, that also produce spasm of the sphincter. In some cases these are the only lesions to be found in the anal canal, and may be overlooked unless a searching examination is made. A digital examination by one not expert will nearly always fail. It is this class of cases in which the sphincter is divulsed by some surgeons, and from which the patients derive little or no benefit.

It may be considered almost an axiom, that there is no abnormally tight sphincter unless there is some pathological lesion causing it, and a divulsion without finding and correcting the lesion will avail little. We cannot, therefore, too strongly emphasise the importance of physicians knowing what these diseased crypts and papillæ are and what they signify, if they are to treat their patients intelligently, and give them the improvement they have a right to expect. I am fully aware that quacks make use of these to mulct their victims of fees, to which they are not entitled, by calling the normal crypts pathological, but it is not of these I speak. The diseased ones give trouble and need as careful and scientific attention as other rectal diseases. I know they are not very frequently found , and many writers ignore them entirely, or only mention them in connection with quacks, and then dismiss the subject.

The crypts should not be operated when normal; if diseased, however and the semilunar valves undermined, the mucous membrane should be split entirely down to the verge of the anus, and a narrow strip cut off each side to prevent reuniting, and thus reproducing the original condition. The operation can be performed under a local anesthetic, when only one or two exist, and when the sphincter is not too tight. When there are several diseased crypts and a tight sphincter, a general anesthetic should be used, a careful preparatory divulsion of the sphincter muscle done, after which the operation should be made in the manner above stated.

Hypertrophied papillæ, which at times stud the mucous membrane on the columns of Morgagni, are usually located near a diseased crypt. These produce an uneasiness, neuralgia, spasm and hypertrophy of the sphincter, while not the least disagreeable sensation is a feeling of incompleteness of bowel movement. This causes the patient to continue straining, and the spasm of the sphincter to be continued until the papillæ resume their normal positions. The only treatment for these is removal, which can be done under a local anesthetic, without confining the patient in bed. Very slight bleeding occurs at the operation.

The thrombotic hemorrhoid is another quite common rectal lesion, that is not understood by some physicians, or if understood is often improperly treated. Time after time patients come to me, saying they have been treated by "Dr. Blank," for a week or ten days, with ice packs, hot poultices, antiphlogistine, ointments, suppositories, lead and opium wash and the like, who have been in bed during this treatment, suffering almost continuously. Repeated attempts have been made to replace these external hemorrhoids inside the anus, both by the medical attendant and the patient. These attempts are worse than useless for they are productive of pain, and only increase the irritation and tension of the parts. Many physicians advise against operation during the acute stage of any form of hemorrhoids, this is contrary to the experience and teaching of the best rectal specialists.

I have been called to consult with some of our best practitioners and found a patient, who has been ill for two or three weeks, suffering intensely from acutely inflamed hemorrhoids.

Many different applications having been made to give relief, without much avail. A rectal surgeon would advise immediate radical operation. The pathology of thrombotic hemorrhoids suggests the only treatment that should be considered —namely, incision. This allows the clots to escape, and at the same time the section of the vein that has ruptured should be removed. Very little, if any pain will follow this operation. The patient can go about his usual work the next day, and the parts will be entirely healed in one to two weeks. This treatment leaves the patient cured without danger of abscess, or even a fistula, which latter can often be traced to these lesions. The so-called palliative treatment leaves the clot to be absorbed, and the ruptured vessel in situ to bleed again, on any severe exertion, or to become infected. It keeps the patient from business, and in severe pain, except for the little relief obtained from the local applications previously mentioned.

Abscess about the rectum or anus is a condition that is deserving of most careful attention. Abscess should be suspected in every case where there is a continuous dull, throbbing pain. Careful search should be made, and any brawny or indurated spot should be incised, even before pus is formed. The habit of many well informed physicians is to poultice, or do other temporizing treatment which will, more likely than not, result in much loss of structure, with burrowing of pus through the loose connective tissue, in all directions. An abscess allowed to go on in this way will almost surely result in a fistula, with all its attendant discomforts and danger.

A fistula should be operated at once, and by so doing, the patient will recover in almost every case, without loss of sphincteric control—one of the worst misfortunes that can befall a human being. The operation for a simple fistula is not at all serious but it is imperative, in all forms of fistula, that all pyogenic membrane should be removed, leaving only the normal tissue, which heals very successfully and rapidly if properly cared for.

Instead of 50 per cent. of failures in operations for fistulæ all except the tubercular variety and many of those should result in complete and permanent cure. In operating for cure of the deeper fistulæ, it is always better, when there is no opening into the bowel, to dissect and remove all pyogenic membrane, and avoid opening into the rectum, thereby more surely preserving the sphincter muscles. It is preferable to do a second operation to perfect a cure than to divide the sphincter unnecessarily. This should be explained to the patient before operation, in order that he may not criticise, if a second operation is found necessary.

Constipation in children is a subject about which I wish to say just a word in closing. The sigmoid in the infant is much too long for the body, and lies across the pelvis to the right side, thus producing an anatomical obstruction, which is relieved as the child grows older, and the sigmoid assumes its normal position. While this condition exists, if a child is constipated, it is my belief that simple saline enemata should be administered, instead of laxative medication.

When physicians feel the necessity of giving something by the mouth, to relieve an habitually costive child, fresh unsalted butter, will in a very large proportion of cases, if given in teaspoonful doses two or three times daily, relieve this troublesome condition. The amount given may be increased or decreased, as the child may need.

800 UNIVERSITY BLOCK.

